# In Vitro Immunomodulation of the Polysaccharides from Yam (*Dioscorea opposita* Thunb.) in Response to a Selenylation of Lower Extent

**DOI:** 10.3390/foods10112788

**Published:** 2021-11-12

**Authors:** Qing-Yun Guan, Ya-Ru Lin, Ling-Yu Li, Zhi-Mei Tang, Xin-Huai Zhao, Jia Shi

**Affiliations:** 1 Key Laboratory of Dairy Science, Ministry of Education, Northeast Agricultural University, Harbin 150030, China; mguan4826@163.com (Q.-Y.G.); lin17853509810@163.com (Y.-R.L.); 17852026615@163.com (L.-Y.L.); 2 School of Biology and Food Engineering, Guangdong University of Petrochemical Technology, Maoming 525000, China; tzm1103@gdput.edu.cn; 3 Research Centre of Food Nutrition and Human Healthcare, Guangdong University of Petrochemical Technology, Maoming 525000, China; 4 Maoming Branch, Guangdong Laboratory for Lingnan Modern Agriculture, Guangdong University of Petrochemical Technology, Maoming 525000, China

**Keywords:** yam polysaccharides, selenium, selenylation, RAW 264.7 macrophages, murine splenocytes, immunomodulation

## Abstract

The immunomodulation of chemically selenylated polysaccharides has been attracting more attention recently, but the corresponding performance of the yam polysaccharides (YPS) with lower selenylation extent remains, thus far, unsolved. In this study, the YPS was selenylated with Na_2_SeO_3_ under acidic conditions generated by HNO_3_ to reach two lower selenylation extents, yielding two selenylated YPSs, namely SeYPS-1 and SeYPS-2 with selenium contents of 715 and 1545 mg/kg, respectively. The results indicated that YPS, SeYPS-1, and SeYPS-2 all had in vitro immuno-modulation when using RAW 264.7 macrophages and murine splenocytes as cell models. In detail, the three polysaccharide samples at dose levels of 5–160 μg/mL showed insignificant cytotoxicity to the macrophages and splenocytes with cell exposure times of 12–24 h, because of the measured values of cell viability larger than 100%. However, Na_2_SeO_3_ at dose levels of 1.3–3.25 μg/mL mostly caused obvious cytotoxic effects on the cells, resulting in reduced cell viability values or cell death, efficiently. The results demonstrated that, compared with YPS, both SeYPS-1 and SeYPS-2 at a lower dose level (5 μg/mL) were more active at promoting phagocytosis activity, increasing the CD4^+^/CD8^+^ ratio of the T-lymphocyte sub-population in the murine splenocyte, improving cytokine secretion, including interleukin-6 (IL-6), IL-1β, and tumor necrosis factor-α in the macrophages, or increasing interferon-γ secretion, but suppressing IL-4 production in the splenocytes. Consistently, SeYPS-2 has more potential than SeYPS-1 at exerting these assessed bioactivities in the cells. Thus, we conclude that a chemical modification of YPS using trace element Se at a lower selenylation extent could bring about higher immunomodulatory activity towards macrophages and splenocytes, while selenylation extent of YPS is a critical factor used to govern the assessed activity changes of YPS.

## 1. Introduction

Selenium (Se) is an essential trace element and is well known for its various biofunctions in the body, especially the fundamental part of the two enzymes, glutathione peroxidase and thioredoxin reductase [1]. In brief, the main biological activities of Se include its ability to prevent diabetes, eliminate free radicals, enhance immunity, and exert anti-cancer and anti-oxidant effects [2]. A previous study revealed that insufficient intake of Se was associated with Keshan disease, immune dysfunction, cancers, stroke, inflammation, and viral diseases [3]. However, Se cannot be synthesized by the body itself; it is obtained mainly through dietary foods [4]. The absorption–utilization of inorganic Se by the human body is quite limited. Thus, it makes sense to find a proven and safe way to enhance Se intake, considering the essential roles of Se in the body [5]. In dietary foods, polysaccharides are also natural food components. Chemically, polysaccharides belong to polymeric carbohydrates that are composed of long chains of monosaccharide units linked by the glycosidic bonds. Meanwhile, non-digestible polysaccharides have attracted more interest in recent years, due to their unique physicochemical structure, biological functioning, and food uses, especially their roles as dietary fibers and the potential to lower the risk of cardiovascular disease, obesity, diabetes, and some cancers [6]. These non-digestible polysaccharides have recently been widely assessed for their biological activities (e.g., immune–modulatory, anti-oxidant, and anti-cancer effects) [7].

Both Se and polysaccharides play an important role in maintaining human health. Thus, it is reasonable to assume that, for example, using a chemical selenylation of polysaccharides, might endow the selenylated polysaccharides with changed biological functions. In the past, various natural selenylated polysaccharides were isolated from mushrooms, bacteria, and plants, and were observed to possess elevated activities [8,9,10,11]. However, selenylated polysaccharides in natural foods are very few, while chemical synthesis is an effective way to generate selenylated polysaccharides. Usually, several selenylation methods using the reaction systems consisting of Na_2_SeO_3_-HNO_3_, H_2_SeO_3_-HNO_3_, Na_2_SeO_3_-CH_3_CO_2_H, or H_2_SeO_3_-CH_3_CO_2_H can be applied to synthesize the selenylated polysaccharides, while the Na_2_SeO_3_-HNO_3_ system was frequently used because of the simple reaction condition and higher selenylation efficiency [12,13]. Clearly, the performed polysaccharide selenylation can lead to obvious changes in these physicochemical properties, such as conformation and water solubility, and can also cause altered immune-modulatory, anti-inflammatory, and anti-tumor activities [14]. More importantly, it was found that polysaccharide selenylation could reduce the limitation of inorganic Se in the food and medical fields, due to the lower toxicity and higher bioactivity of organic Se [1,15].

The immune system is regarded as a biological network that is comprised of immune organs, cells, and molecules. It plays a critical role in detecting and responding to the stimuli induced by toxins, viruses, parasites, and cancer cells. This system coordinates with other systems in the body to maintain the vital stability of the internal environment and physiological balance [16,17]. Previous results have shown that immune cells (especially the macrophages and splenocytes) could be influenced by plant polysaccharides [18,19]. Functionally, the macrophages and splenocytes can secret these bioactive molecules, such as interleukins, interferon, and tumor necrosis factor to regulate the activity of the immune-related cells. In addition, the macrophages are considered as “the interface” of the innate and adaptive immunity, and can play a role in clearing damaged or dead cells through these pathways. Furthermore, the mitogen-activated protein kinase (MAPK) and nuclear factor (NF)-κB-signaling pathways are the most important signaling pathways involved in the macrophages [20]. The splenocytes mainly include T-lymphocytes, B-lymphocytes, macrophages, etc. Thus, the immune functions of the monocyte–macrophages and lymphocytes are usually chosen to reflect the cellular and humoral immune levels of the body. It is known that polysaccharides can exert their immunomodulation in the body, mainly through nonspecific immunity, resulting in the enhanced phagocytic ability of phagocytes, promoted production of immune cytokines, such as IL-6, IL-1β, and TNF-α, improved activities of macrophages, NK cells, and dendritic cells, together with enhanced secretion of antibodies [21]. In addition, some plants, which contain natural polysaccharides, are used in traditional medicines because of their non-specifically stimulating activity towards immune cells [22].

Yam (*Dioscorea opposita* Thunb.) is widely cultured in East Asia, especially China, as a food crop and traditional Chinese medicine for centuries. Additionally, the Chinese yam is the only edible yam species that can be cultivated in temperate regions, such as Europe [23]. The tubers of this crop contain many bioactive compounds, such as mucilage polysaccharides and steroidal sapogenins [24]. Yam polysaccharides are considered important components that provide both biological and physicochemical properties and, thus, can be isolated and used as health-promoting ingredients in foods [25,26]. The yam polysaccharides have also been assessed for their chemical and biological properties, such as immune-modulatory, anti-oxidative, anti-inflammatory, and anti-tumor effects. Chemically, the main monosaccharide compositions of yam polysaccharides were detected to be glucose, mannose, xylose, arabinose, and rhamnose [27,28], while these chemical characteristics, at times, had some variability, because of the specific yam sources and the used extraction and purification methods [29]. In addition, it is now agreed that yam polysaccharides could promote macrophage phagocytosis, lymphocyte proliferation, and natural killer (NK) cell activity [30]. Meanwhile, the yam polysaccharides were also able to scavenge hydroxyl and superoxide anion radicals, and decrease the LPS-induced NO production and expressions of iNOS and COX-2 in RAW 264.7 macrophages [31,32]. More importantly, the immunomodulatory effects of polysaccharides have been considered to associate with their chemical features, including molecular weight, monosaccharide composition, glycosidic linkage, as well as chain conformation [29].

In this study, the soluble yam polysaccharides were extracted from the edible parts of fresh yam by water. Afterwards, the yam polysaccharides were selenylated into two selenylation extents using the Na_2_SeO_3_-HNO_3_ method. The in vitro immune–modulatory activities of the selenylated polysaccharides were then evaluated using the RAW 264.7 macrophages, with murine splenocytes as cell models and unreacted yam polysaccharides as a control. Potential cytotoxicity of the selenylated polysaccharides on the cells was firstly clarified, while their impacts on macrophage phagocytosis, stimulation index of splenocytes, T-lymphocyte subpopulations, and cytokine secretions of IL-6, IL-1β, TNF-α, IL-4, and IFN-γ were also measured. This study aimed to reveal whether a chemical selenylation of the yam polysaccharides might cause immune–modulatory changes, especially whether selenylation extent could govern the polysaccharide activity to the two cells.

## 2. Materials and Methods

### 2.1. Materials and Reagents

The fresh yam, cultured in Jiaozuo City, Henan province of China, was purchased from a local market in Harbin, Heilongjiang province. Fetal bovine serum (FBS) was purchased from Thermo Fisher Scientific Inc. (Cleveland, OH, USA), while Dulbecco’s Modified Eagle Medium (DMEM) and RPMI-1640 were obtained from HyClone Co. (Logan, UT, USA). The phosphate-buffered saline (PBS) and α-amylase were bought from Solarbio Science and Technology Co., Ltd. (Beijing, China). The alkaline protease was bought from Beijing Aoboxing Biotechnologies, Inc. (Beijing, China). The phycoerythrin (PE)-conjugated anti-mouse CD8a^+^ antibodies, and fluorescein isothiocyanate (FITC) anti-mouse CD4^+^ antibodies were purchased from Miltenyi Biological Technology Co. Ltd. (Bergisch Gladbach, Cologne, Germany). Concanavalin A (Con A), lipopolysaccharide (LPS), and 3-(4,5-dimethyl-2-thiazolyl)-2,5-diphenyl tetrazolium bromide (MTT) were bought from Sigma Aldrich (St. Louis, MO, USA). The trypan blue and neutral red were bought from AMRESCO, Inc. (Los Angeles, CA, USA). The Cell Counting Kit-8 (CCK-8) was bought from Meilun Biological Technology Co. Ltd. (Dalian, China). All cytokine kits were provided by Wuhan Boster Biological Technology Co., Ltd. (Wuhan, China). Other chemical reagents used were of analytical grade. Ultrapure water generated from Milli-Q Plus (Millipore Corporation, New York, NY, USA) was used in cell experiments.

### 2.2. Animals and Cells

Female BALB/c mice (6–8 weeks old) were bought from Beijing Vital River Experimental Animal Technical Co. Ltd. (Beijing, China). All animal experiments were consistent with the protocols of the Animal Care and Use Committee of Northeast Agricultural University (Harbin, China).

RAW 264.7 macrophages were bought from the Chinese Academy of Sciences (Shanghai, China). The cells were maintained in the DMEM supplemented with 10% FBS and 100 U/mL penicillin/streptomycin at 37 °C with a humidified atmosphere of 5% CO_2_ as recommended by the cell supplier. The cells were maintained by subculturing every 2 days. The passages of 5–10 were used in this study.

### 2.3. Preparation and Assay of Yam Polysaccharides and Selenylated Polysaccharides

The preparation of yam polysaccharides (YPS) followed a previous study with a minor modification [30]. In brief, the fresh yam was washed, peeled, cut into small pieces, mashed, and homogenized by a blender. The mashed yam was mixed with distilled water at the mass ratio of 1:10, added α-amylase (20 U/mL), kept at 80 °C for 4 h, and then centrifuged at 8000× *g* for 15 min to collect the supernatant. Afterwards, alkaline protease (100 U/mL) was added to the supernatant, kept at 55 °C for 6 h to hydrolyze the proteins, concentrated into 1/8 of the original volume, and mixed with three volumes of anhydrous ethanol at 4 °C for 24 h. The collected precipitates (i.e., YPS) were washed by anhydrous ethanol three times, and then freeze-dried.

The YPS were selenylated using Na_2_SeO_3_-HNO_3_ as in the previous studies with slight modification [10,33]. In brief, 500 mg YPS powder was added into 8 mL HNO_3_ (0.5%, V/V) and stirred for 0.5 h at 20 °C, added with 25–50 mg of Na_2_SeO_3_, and kept at 75 °C for 8 h. After the reaction, the whole system was cooled to 20 °C, treated with three volumes of anhydrous ethanol. The collected sediments were washed three times by anhydrous ethanol and freeze-dried. Two selenylated yam polysaccharides (namely SeYPS-1 and SeYPS-2) were thus obtained. In addition, YPS and Na_2_SeO_3_ were mixed without HNO_3_ and treated as above. The obtained YPS was used as a control in Se detection.

The Se contents of YPS, SeYPS-1, and SeYPS-2 were measured by an inductively coupled plasma-mass spectrometer (Agilent Technologies, Santa Clara, CA, USA) as previously described [34].

### 2.4. Assays of Macrophages Viability

Cell viability was assessed using the MTT method as previously described [20]. In brief, the cells (2 × 10^5^ cells/mL, 100 μL/well) were seeded into the 96-well plates and incubated for 4 h. After discarding the medium, 100 μL of YPS, SeYPS-1, and SeYPS-2 at various dose levels (5–160 μg/mL) were added into each well to treat the cells for 12–24 h. Na_2_SeO_3_ at 1.3–3.25 μg/mL was also used to treat the cells similarly. Afterwards, the medium was discarded and 100 μL of MTT solution was added into each well to incubate for 4 h. After supernatant removal, 100-μL dimethyl sulfoxide (DMSO) was added into each well. The OD values at 490 nm were measured using a microplate reader (Bio-Rad Laboratories, Hercules, CA, USA). The values of cell viability were calculated according to the previous study [35], while the control cells were designed with 100% viability.

### 2.5. Assays of Phagocytic Activity and Cytokine Secretion

The phagocytic activity was measured using the neutral red method as previously described [30]. Briefly, the cells (2 × 10^5^ cells/mL, 200 μL/well) were seeded into the 96-well plates and incubated for 4 h. After discarding the supernatants, 200 μL/well of the samples at different dose levels (5–80 μg/mL) were added to treat the cells for 24 h. After the medium was discarded, 100 μL of 1% neutral red solution was added into each well to incubate the cells for 30 min. After supernatant removal, the cells were washed five times by the PBS (10 mmol/L, pH 7.2), and then 200 μL of lysing solution (1% acetic acid: ethanol = 1:1, *V/V*) was added to treat the cells for 2 h. The absorbance at 540 nm was measured by the microplate reader. The phagocytosis index (PI) was thus calculated as: PI = Abs (sample)/Abs (blank).

The secretion levels of cytokines were assessed following the protocols provided by the kit manufacturers. In brief, the cells (2 × 10^5^ cells/mL, 2 mL/well) were seeded into the 6-well plates and incubated for 4 h. After the medium removal, 2 mL of the samples at dose levels of 5–80 μg/mL were added into each well to treat the cells for 24 h. Afterwards, the supernatants were collected using a centrifugation at 500× *g* for 20 min and assayed for the secretion levels of IL-6, IL-1β, and TNF-α using the respective ELISA kits.

### 2.6. Preparation of Splenocytes and Assays of Splenocytes Proliferation

The splenocytes were isolated from female BALB/c mice as previously described [36]. In detail, the mice were sacrificed by cervical dislocation and submerged in 75% alcohol for 5 min. The spleen was removed from the abdominal cavity in the sterile environment, and then placed in a sterilized plate containing the cold Hank’s balanced salt mixture. The splenocytes were thus obtained after the spleen was minced and passed through a 200-mesh sieve. Afterwards, the cells were washed by the PBS and centrifuged at 170× *g* for 5 min to harvest. After supernatant removal, the cells were treated by 5 mL lysis buffer for 3 min, centrifuged at 170× *g* for 5 min, washed by the PBS, and centrifuged again. The collected cells were suspended in the medium. Trypan blue was used to detect the proportion of living cells to ensure cell viability larger than 98%.

Splenocyte proliferation was assessed according to a previous study [37]. In brief, the splenocytes (1 × 10^6^ cells/mL, 100 μL/well) were inoculated into the 96-well plates, added with 100 μL/well of the samples at 5–80 μg/mL, and then incubated for 48 h. The same experiments were performed in the presence of Con A (5 μg/mL) or LPS (10 μg/mL), respectively. After discarding the medium, 100 μL of CCK-8 solution was added into each well while the cells were incubated for 4 h. The absorbance at 450 nm was measured at the microplate reader. The obtained data were used to calculate stimulation index (SI) values as previously described [37].

### 2.7. Assays of T-Lymphocyte Subpopulations and Cytokine Secretion

The T-lymphocyte subpopulations were measured following a previous study [36]. Briefly, 1.0 mL splenocytes (1 × 10^6^ cells/mL) were seeded into 12-well plates and added with 0.5 mL of the samples (dose levels of 5–80 μg/mL) together with Con A (5 μg/mL) to stimulate the cells for 48 h. Then, the cells were centrifuged at 170× *g* for 5 min, washed twice by the PBS, and suspended into the PBS to reach 2 × 10^6^ cells/mL. The aliquot of the suspensions containing 1 × 10^6^ cells was stained by 10-μL PE-conjugated anti-mouse CD8a^+^ and 10 μL FITC-conjugated anti-mouse CD4^+^ antibodies at 4 °C for 30 min in the dark, and then passed through a 300-mesh sieve. The splenocytes (1 × 10^4^ cells) were counted and analyzed by a flow cytometer (Type BD FACS Aria II, BD Bioscience, Franklin Lakes, NJ, USA).

The cytokine secretion was measured following the protocols provided by the kit manufacturers. In brief, the splenocytes (1 × 10^6^ cells/mL, 1.0 mL/well) were seeded into the 12-well plates, followed by a treatment of the samples (0.5 mL, dose levels of 5–80 μg/mL) for 48 h. Afterwards, the supernatant was collected by a centrifugation at 500× *g* for 20 min to assay the levels of IL-4 and IFN-γ, using the respective ELISA kits.

### 2.8. Statistical Analysis

All experiments and assays were performed in triplicates, while the results were expressed as means ± standard deviations. The differences between the mean values were analyzed via one-way ANOVA analysis of variance followed by Duncan’s multiple range test (*p* < 0.05). All statistical analyses were performed using SPSS (SPSS-16, Chicago, IL, USA).

## 3. Results

### 3.1. Cytotoxicity of the Polysaccharide Samples and Na_2_SeO_3_ to Macrophages

The prepared YPS in this study had a saccharide content of 897.3 g/kg (dry basis), determined by the classic phenol-H_2_SO_4_ method. Meanwhile, Se contents of the control YPS, SeYPS-1, and SeYPS-2 were measured to be 57, 715, and 1545 mg/kg, respectively, indicating that 658 (SeYPS-1) and 1488 mg Se (SeYPS-2) were attached into the YPS of 1 kg. Both SeYPS-1 and SeYPS-2 had much higher Se content than YPS, implying they might have increased or decreased activities than YPS in the cells. SeYPS-2 showed much higher Se content (near two folds) than SeYPS-1; thus, a data comparison of their activity differences would clarify briefly how the achieved selenylation extent impacted the assessed activities of the selenylated YPS towards the cells.

**Figure 1:**
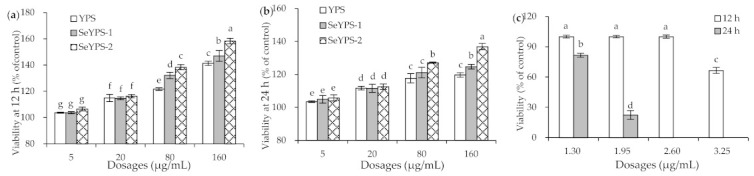
The viability values of the macrophages exposed to YPS, SeYPS-1, SeYPS-2 (**a**,**b**) or Na_2_SeO_3_ (**c**) for 12 or 24 h. Different letters above the columns indicate that the one-way ANOVA of the mean values differs significantly (*p* < 0.05).

When the macrophages were exposed to the three polysaccharide samples at 5, 20, 80, and 160 µg/mL for 12–24 h, the cells obtained growth promotion dose-dependently (Figure 1a,b). Overall, the three samples did not show any cytotoxicity on the cells, because the measured values of cell viability were all larger than 100% (i.e., from 103.5% to 158.3%). Meanwhile, the two selenylated polysaccharides (especially SeYPS-2) had higher growth promotion than YPS on the cells, confirming that the performed chemical selenylation endowed YPS with enhanced bioactivity to the cells. However, when Na_2_SeO_3_ alone was used at 1.3–3.25 µg/mL to treat the cells for 12–24 h, it mostly exerted cytotoxicity on the cells (Figure 1c). The shorter treatment time of 12 h and higher Na_2_SeO_3_ dose of 3.25 µg/mL led to reduced cell viability to 66.5%, while the cell treatment time of 24 h and higher Na_2_SeO_3_ doses of 2.6–3.25 µg/mL even resulted in total cell death (cell viability close to zero). This fact meant that inorganic Se (e.g., in the form of Na_2_SeO_3_) is very toxic to the macrophages.

To compare the immune-regulatory potentials of these polysaccharide samples further, the dose levels of 5, 20, and 80 µg/mL were selected in cell experiments.

### 3.2. Phagocytic Activity and Cytokine Production of the Macrophages in Response to the Polysaccharide Samples

**Figure 2:**
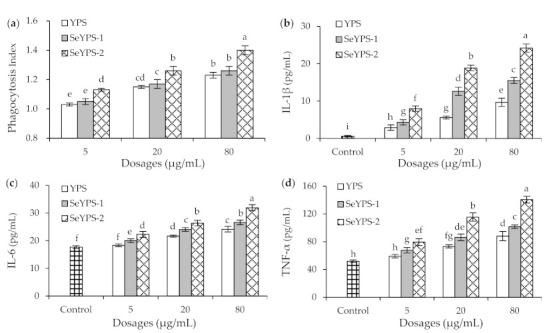
Phagocytosis index (**a**) and secretion levels of IL-1β (**b**), IL-6 (**c**), and TNF-α (**d**) of the macrophages treated by SYP, SeYPS-1, and SeYPS-2 for 24 h. Different letters above the columns indicate that the one-way ANOVA of the mean values differs significantly (*p* < 0.05).

When the macrophages were stimulated by YPS, SeYPS-1, and SeYPS-2 at the dose levels of 5, 20, and 80 µg/mL for 24 h, their phagocytosis activity was enhanced in a dose-dependent manner (Figure 2a). All the three polysaccharide samples showed a stimulation on the macrophages to increase their phagocytosis, because all measured PI values were larger than 1. Compared with SYP, the two selenylated polysaccharides (especially SeYPS-2) exhibited a higher potential to enhance macrophage phagocytosis (PI values 1.03–1.23 vs. 1.05–1.40). Meanwhile, SeYPS-2 always was more active than SeYPS-1 to enhance macrophage phagocytosis (PI values 1.13–1.40 vs. 1.05–1.26). All of these results demonstrated that the chemical selenylation of YPS brought about a higher activity to promote macrophage phagocytosis while a higher selenylation extent led to much higher activity for the selenylated YPS.

The results also indicated that YPS, SeYPS-1, and SeYPS-2 could improve the immune status of macrophages efficiently, because they showed the ability to promote the secretion of three immune cytokines dose-dependently (Figure 2b–d). In detail, when the macrophages were exposed to these samples, IL-1β secretion levels were increased from 0.6 (control cells) to 2.9–24.2 pg/mL, while IL-6 secretion levels were enhanced from 17.7 (control cells) to 18.4–31.9 pg/mL. At the same time, the secretion levels of TNF-α were also elevated from 51.8 (control cells) to 59.1–140.5 pg/mL. Consistently, both SeYPS-1 and SeYPS-2 had more potential than YPS at each assessed dose level to favor the secretion of the three cytokines IL-1β, IL-6, and TNF-α, while SeYPS-2 at all cases caused more cytokine secretion than SeYPS-1 did. Thus, it is concluded that the performed selenylation endowed the selenylated YPS with enhanced activity to stimulate the macrophages for higher production of IL-1β, IL-6, and TNF-α, while higher incorporation of Se into YPS (i.e., higher selenylation extent) yielded higher stimulation on the macrophages.

### 3.3. Effects of the Polysaccharide Samples on Splenocyte Proliferation

**Figure 3:**
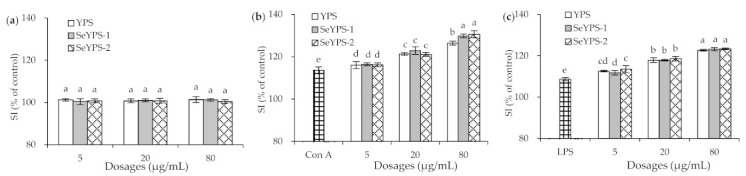
The measured SI values of the splenocytes exposed to YPS, SeYPS-1, and SeYPS-2 for 48 h without the mitogen (**a**) and accompanied with Con A (**b**) or LPS (**c**). Different letters above the columns indicate that the one-way ANOVA of the mean values differs significantly (*p* < 0.05).

When the splenocytes were incubated with YPS, SeYPS-1, and SeYPS-2 without the mitogen for 48 h, no significant change in SI value was observed (Figure 3a). These results demonstrated that no sample had obvious cytotoxicity or other effects on the cells (*p* > 0.05), because the measured SI values were all close to 100%. However, when Con A and LPS were used separately to stimulate the splenocytes, the cells were detected with much-enhanced SI values (Figure 3b,c), indicating that both Con A and LPS induced cell proliferation. With the mitogen stimulation, the samples at dose levels of 5–80 µg/mL also enhanced SI values dose-dependently. Using the Con A stimulation, the cells incubated with YPS, SeYPS-1, and SeYPS-2 showed higher SI values than the model cells (116.0–130.6% vs. 113.6%). While the cells were stimulated by LPS, the elevated SI values were increased from 108.7% (model cells) to 111.8–123.4%. In total, SeYPS-1 and SeYPS-2 were mostly not different at these dose levels in enhancing SI values; more importantly, YPS mostly exerted the same activity as SeYPS-1 and SeYPS-2. Thus, it is regarded that the performed selenylation might not alter the proliferation of YPS on the Con A- or LPS-stimulated splenocytes. Thus, it was necessary to clarify whether the samples had the ability to impact cytokine secretion and T-lymphocyte subpopulations in the splenocytes.

### 3.4. Effect of the Polysaccharide Samples on Cytokine Production and T-Lymphocyte Subpopulations in Splenocytes

**Figure 4:**
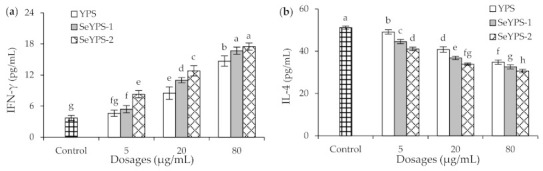
The production of IFN-γ (**a**) and IL-4 (**b**) in the splenocytes treated by YPS, SeYPS-1, and SeYPS-2 for 48 h. Different letters above the columns indicate that the one-way ANOVA of the mean values differs significantly (*p* < 0.05).

When the splenocytes were treated by YPS, SeYPS-1, and SeYPS-2 at dose levels of 5–80 µg/mL for 48 h, the secretion levels of two immune cytokines, namely IFN-γ and IL-4, were increased or decreased by the samples clearly (Figure 4). Overall, all assessed samples showed the capacity to enhance IFN-γ secretion but suppress IL-4 secretion dose-dependently. Specifically, the detected secretion levels of IFN-γ were increased from 3.7 (control cells) to 4.6–17.5 pg/mL, while those of IL-4 decreased from 51.1 (control cells) to 30.6–49.1 pg/mL. In addition, the selenylated polysaccharide samples SeYPS-1 and SeYPS-2 had higher potential than YPS to modulate cytokine secretion in the splenocytes, while SeYPS-2 was mostly more effective than SeYPS-1 to perform this biofunction. All results consistently pointed out that the conducted selenylation endowed YPS with higher potential to modulate the immune status of the splenocytes through increasing IFN-γ secretion but decreasing IL-4 secretion, while higher selenylation extent contributed for this mentioned cytokine modulation.

**Figure 5:**
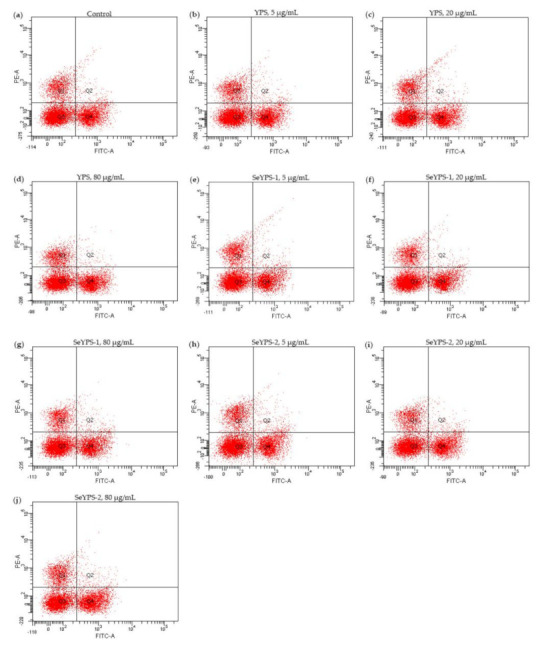
The analyzed results for T-lymphocyte subpopulations by flow cytometry for the control splenocytes (**a**), or those treated by YPS (**b**–**d**), SeYPS-1 (**e**–**g**), and SeYPS-2 (**h**–**j**) at different dosages.

If the splenocytes were incubated with the samples at the dose levels of 5–80 µg/mL and Con A (5 µg/mL) for 48 h, the CD4^+^/CD8^+^ ratios of the treated cells were also enhanced dose-dependently (Figure 5). The detailed results are listed in Table 1. The data indicated that the samples mostly had a slightly higher ability to increase the CD4^+^/CD8^+^ ratio of T-lymphocytes, especially at the dose levels of 20 and 80 µg/mL. Meanwhile, the two selenylated polysaccharide samples (especially SeYPS-2) were more able than YPS to increase the CD4^+^/CD8^+^ratio. Thus, it was regarded that the performed YPS selenylation also conferred SeYPS-1 and SeYPS-2 with higher potential to mediate T-lymphocyte subpopulations in the stimulated splenocytes. In other words, the YPS selenylation could increase the activity of YPS to modulate the immune status of the splenocytes.

## 4. Discussion

In general, the immunomodulatory property of natural polysaccharides is associated with their unique chemical characteristics including molecular weights, spatial configuration, and monosaccharide compositions [38,39,40]. Therefore, the chemical modification of polysaccharides (e.g., selenylation) could influence these characteristics significantly [12,41]. For example, when the garlic polysaccharides were selenylated by the Na_2_SeO_3_-HNO_3_, H_2_SeO_3_-HNO_3_, Na_2_SeO_3_-CH_3_CO_2_H, H_2_SeO_3_-CH_3_CO_2_H, and SeCl_2_O methods, respectively, the measured Se contents of these products were up to 6210–29,400 mg/kg. More importantly, the results also confirmed that using the Na_2_SeO_3_-HNO_3_ method caused the highest selenylation efficiency for the target garlic polysaccharides [10]. If the *Atractylodes macrocephala* polysaccharides were selenylated by the Na_2_SeO_3_-HNO_3_ method, the bound Se contents were measured to be 6120–12,230 mg/kg [42]. Furthermore, when the pectic polysaccharides from *Ulmus pumila* L. were selenylated using Na_2_SeO_3_-HNO_3_, the bound Se contents would range from 3240 to 13,190 mg/kg [43]. All these mentioned results confirmed that the Na_2_SeO_3_-HNO_3_ method could be used to selenylated polysaccharides effectively, yielding a higher Se level of the selenylated polysaccharides. However, from a chemical perspective, it is reasonable to assume that the chemical characteristics of polysaccharides may be altered under the used reaction conditions; for example, the glycosidic bonds in polysaccharides might be broken during the chemical selenylation. Consequentially, the polysaccharides have undesired degradation. On the other hand, the recommended nutrient intake (RNI) of Se is about 50 µg/d for adults, and the specific intake depends on the health status of the body and environmental differences [44]. Excessive Se intake (especially in the inorganic form) is considered to bring about toxicological effects by causing cognitive and behavioral impairments to humans [45]. Thus, it is necessary to selenylated polysaccharides under controllable, reliable, and effective conditions, considering that the selenylated polysaccharides might be feasible food supplements. This means that the preparation and evaluation of the selenylated polysaccharides with lower Se content are also important. In the present study, a chemical selenylation at a lower extent was thereby applied to the YPS.

The chemical selenylation could endow the polysaccharides with greater bioactivities including immunomodulation, anti-oxidation, anti-inflammation, and anti-cancer effect [12]. It was proved that the selenylated *Cordyceps militaris* polysaccharides had a stronger anti-tumor effect on hepatocellular carcinoma HepG-2 cells and lung adenocarcinoma A549 cells by inhibiting the cell proliferation [46], while the selenylated *Ulmus pumila* L. pectic polysaccharides possessed higher anti-oxidant activity to reduce Fe^3+^ or scavenge various radicals [47]. Meanwhile, the selenylated sweet potato polysaccharides could scavenge free radicals much efficiently, and also could inhibit tumor growth, increase the secretion levels of IL-2 and TNF-α but decrease the serum vascular endothelial growth factor (VEGF) level in the murine H22 hepatoma mice model [48]. Furthermore, the selenylated lentinan was more effective to inhibit the progress of inflammation and fibrosis, through enhancing superoxide dismutase and glutathione peroxidase activities, preventing lipid peroxidation, and decreasing the secretion levels of TNF-α and IL-1β, as well as pancreatic hydroxyproline in the 3,5-diethoxycarbonyl-1,4-dihydrocollidine (DDC) mouse model [49]. It is well agreed that macrophages are professional phagocytes, and phagocytosis signifies the first and unique process in the immune response [50]. In addition, the proliferation of splenocytes reflects the status of the immune response level, while the reactive differentiation of T-lymphocytes is considered a key part of adaptive immunity [51]. CD4^+^ (T helper cells) and CD8^+^ (cytotoxic T-lymphocytes) are two of the main intensively studied subpopulations. CD4^+^/CD8^+^ ratio is regarded as a marker for both immune senescence and immune activation [52]. It was reported that the selenylated *Lilium davidii* var. *unicolor Salisb* polysaccharides had immunomodulation by up-regulating phagocytosis and IL-1β secretion in the macrophages [14]. Moreover, the selenylated *Chuanminshen violaceum* polysaccharides could promote T-lymphocyte proliferation, enhance the activities of NK cells and cytotoxic T-lymphocytes, and promote the secretion of IL-4, IL-2, and IFN-γ in CD4^+^ T cells [53]. The present study also confirmed that the selenylated YPS had enhanced immune functions in the two immune cells by enhancing macrophage phagocytosis, increasing the CD4^+^/CD8^+^ ratio of T-lymphocytes, as well as mediating the secretion levels of the assessed cytokines. Thus, the present study shares a conclusion similar to these previous studies.

Food components, after their chemical modifications, generally have changed biofunctions in cells. In detail, the tryptic caseinate hydrolysates with oligochitosan glycation had higher potential to increase the secretion levels of immunoglobulins, enhance spleen and thymus indices, and promote splenocyte lymphocyte proliferation and NK cell activity in the BALB/c mice [54]. When YPS was efficiently hydrolyzed by inorganic acid, the hydrolyzed products totally lost their immunomodulation; however, thermal treatment of YPS totally led to unchanged immunomodulatory activities [55]. Polyphenols are unstable upon heat treatment and thus perform various chemical reactions, including oxidation, ring cleavage, and others. The heated polyphenols thereby exert changed bioactivities. Specifically, the heated apigenin and luteolin in the human cervical cancer Hela cell model showed decreased activities in growth inhibition, apoptosis induction, DNA damage, and intracellular reactive oxygen species (ROS) generation [56], while the oxidized and heated flavonols showed weaker anti-proliferation, apoptosis induction, and membrane disruption than native flavonol counterparts in the human colon cancer HCT-116 cells [57]. It is reasonable that the selenylated YPS had different chemical features than the unmodified YPS and, thus, were detected with different bioactivities in the macrophages and splenocytes.

According to a previous study, the longan polysaccharides enhanced macrophage activation partly via the TLR2- and TLR4-mediated MyD88/IRAK4-TRAF6 signaling pathways [58]. It was also reported that the *Dendrobium huoshanense* polysaccharides activated the macrophages by direct binding with TLR4 to trigger TLR4 signaling pathways [59], while the *Atractylodis macrocephalae Koidz.* polysaccharides could induce splenocytes activation partly via the TLR4-independent MAPKs and NF-κB signaling pathways [60]. At the same time, the *Ganoderma atrum* polysaccharides induced the activation of spleen lymphocytes via the cooperatively regulated Ca^2+^/CaN/NFAT/IL-2 and PKC/NFAT/IL-2 pathways [61]. However, we now know little about how the selenylated YPSs affected the two immune cells. Thus, a future investigation is necessary to reveal the involved signaling pathways mediating the activation of SeYPS-1/-2 on the macrophages and splenocytes, and whether the higher selenylation extent yields much activity changes. At the same time, the in vivo activity of SeYPS-1/-2 also needs to be clarified in the future.

## 5. Conclusions

The present study highlighted that a chemical selenylation of yam polysaccharides by Na_2_SeO_3_-HNO_3_, to lower selenylation extent, might cause a covalent connection of a trance nutrient Se into the polysaccharide molecules, and yield effectively higher immune–modulatory functioning in RAW 264.7 macrophages and murine splenocytes. Compared with the unmodified yam polysaccharides, the selenylated polysaccharides were more active at enhancing phagocytic activity and cytokine secretion, including IL-6, IL-1β, and TNF-α in the macrophages, were able to promote the stimulation index of the Con A- or LPS-stimulated splenocytes, or had a higher ability to mediate the CD4^+^/CD8^+^ ratio in the T-lymphocytes and secretion of IL-4 and IFN-γ in the splenocytes. Moreover, the higher selenylation extent of yam polysaccharides brought about greater activity change in the targeted cells. Thus, it is concluded that this chemical selenylation is a possible approach to incorporate inorganic nutrient Se into polysaccharide molecules and simultaneously enhance the immune activity of polysaccharides towards the two immune cells, efficiently. Whether polysaccharide selenylation of higher selenylation extent has a similar or different impact on these assessed activities needs further investigation. In addition, it is strongly encouraged in agricultural production to cultivate the yam in Se-rich soil, aiming to obtain yam polysaccharides with higher organic Se content and, thereby, higher health benefits to the body.

## Figures and Tables

**Table 1:** The measured T-lymphocyte sub-populations (%) in the Con A-stimulated splenocytes as affected by YPS, SeYPS-1, and SeYPS-2 at 5, 20, and 80 µg/mL for 48 h.

Index	Cell Group	Dose Level of Polysaccharide Sample (μg/mL)		
		5	20	80
CD4^+^	Control	29.4 ± 1.5		
	YPS	29.9 ± 2.5	30.7 ± 2.8	30.6 ± 4.4
	SeYPS-1	29.3 ± 1.2	29.0 ± 2.4	29.7 ± 1.3
	SeYPS-2	29.8 ± 0.6	29.6 ± 1.4	30.5 ± 3.3
CD8^+^	Control	16.4 ± 1.6		
	YPS	15.4 ± 3.5	15.4 ± 4.0	15.2 ± 2.4
	SeYPS-1	14.9 ± 1.6	14.5 ± 2.2	13.9 ± 1.0
	SeYPS-2	14.4 ± 0.8	13.9 ± 0.8	13.6 ± 1.2
CD4^+^/CD8^+^	Control	1.99 ± 0.02		
	YPS	1.94 ± 0.14	2.10 ± 0.44	2.12 ± 0.22
	SeYPS-1	1.98 ± 0.15	2.17 ± 0.02	2.13 ± 0.04
	SeYPS-2	2.07 ± 0.20	2.17 ± 0.09	2.24 ± 0.04

## Data Availability

All data are contained within the article.
